# Characterization and Separation of Live and Dead Yeast Cells Using CMOS-Based DEP Microfluidics

**DOI:** 10.3390/mi12030270

**Published:** 2021-03-06

**Authors:** Honeyeh Matbaechi Ettehad, Christian Wenger

**Affiliations:** 1 IHP—Leibniz-Institut für Innovative Mikroelektronik, Im Technologiepark 25, 15236 Frankfurt (Oder), Germany; wenger@ihp-microelectronics.com; 2 BTU Cottbus-Senftenberg, 03046 Cottbus, Germany

**Keywords:** cell characterization, cell separation, dielectrophoresis (DEP), interdigitated electrodes (IDEs), microfluidics, CMOS-based lab-on-a-chip

## Abstract

This study aims at developing a miniaturized CMOS integrated silicon-based microfluidic system, compatible with a standard CMOS process, to enable the characterization, and separation of live and dead yeast cells (as model bio-particle organisms) in a cell mixture using the DEP technique. DEP offers excellent benefits in terms of cost, operational power, and especially easy electrode integration with the CMOS architecture, and requiring label-free sample preparation. This can increase the likeliness of using DEP in practical settings. In this work the DEP force was generated using an interdigitated electrode arrays (IDEs) placed on the bottom of a CMOS-based silicon microfluidic channel. This system was primarily used for the immobilization of yeast cells using DEP. This study validated the system for cell separation applications based on the distinct responses of live and dead cells and their surrounding media. The findings confirmed the device’s capability for efficient, rapid and selective cell separation. The viability of this CMOS embedded microfluidic for dielectrophoretic cell manipulation applications and compatibility of the dielectrophoretic structure with CMOS production line and electronics, enabling its future commercially mass production.

## 1. Introduction

Characterization and manipulation of biological cells are critical in biomedical and environmental applications. Cells contain crucial information about biological processes and environmental situations [1]. Cell separation, a subcategory of cell manipulation, is vital in clinical applications. Therefore, the secondary aim of a full sample-to-result lab-on-a-chip (LoC) relies on its capability to separate or isolate a cell kind from a cell mixture. For example, separating and sorting live and dead cells is crucial for early stage disease diagnosis [2,3]. Microfluidic LoC devices offer significant benefits in terms of efficiency, accuracy, and cost compared to other macroscopic counterparts [4,5,6]. There are many ways to characterize and separate cells using a microfluidic device, such as filtration, centrifugation, optical and magnetic tweezers, chromatography, dielectrophoresis (DEP), etc., [7,8,9,10,11,12,13,14,15,16,17,18]. However, among these techniques, DEP has been widely utilized in microfluidics for such biomedical applications [12,13,19,20,21,22,23,24,25,26,27,28]. This electrode-based AC electrokinetic technique requires label-free sample preparation, making the sample processing very simple. It also proposes excellent advantages regarding cost, operational power, efficiency, speed, sensitivity, selectivity, and ease of electrode integration with the device architecture [29]. The combination of microfluidic and electrokinetic actuation platforms leads to a promising direction towards complex sample handling procedures.

The advent of complementary metal-oxide-semiconductor (CMOS) technology and its integration with the LoC system enabled the fully functional sample-to-result LoC devices, which aids in device portability even out of the laboratory or hospitals. CMOS integrated LoC device can manage the data from microfluidics, sensors, and actuators. The integration of microfluidic channel with CMOS can scale down multiple-stage laboratory procedures in a single chip and process micro and nano-liters samples within a fully isolated manner. Implementing CMOS logic into practice is simple and consumes little to no current in an idle state [30]. Based on the More Than Moore approach, the CMOS devices’ size is getting smaller and smaller. This approach, on the one hand, allows more functionality to fit in a smaller area. On the other hand, it can provide the right platform for miniaturized hand-held and versatile microfluidic devices [30]. Thus far, a lot of setups have been introduced for such applications. These setups are often bulky and desktop-sized that limits the functionality of the microfluidics to only laboratory-based applications. However, the lack of a promising miniaturized system remains a challenge, and realizing a fully functional device is under research.

This work aims toward the realization of the completely CMOS miniaturized portable and versatile biomedical devices capable of performing various processing laboratory steps (cell preparation, characterization, detection, and separation) using a single device. Monolithic integration of this device and its potentiality for both sample preparation and analyzation on a single platform, as well as operational simplicity and employing a single set of electrodes, offers excellent benefits from a cost and commercialization perspective. The device also provides significant potentialities in terms of sensing, read-out the trapped cells, and performing both sample preparation and analysis (manual or automated using machine learning) on a single device. The simplicity of the device design and its extended functionality for separation as well as detection applications, combined with its flexibility to use different cell types, can place this LoC device among the prominent systems developed to date for commercialization. There are exciting possibilities for the future development of this LoC platform, e.g., the development of multi-functional LoC to be used for clinical diagnostics or as part of the point of care (PoC) or near-patient portable testing systems outside hospitals. The state-of-the-art of this work relies on employing the simplest, most convenient, reliable, and accurate methods and geometries reported so far in one platform.

The combination of CMOS-based microfluidics with dielectrophoresis (DEP) has been demonstrated as one of the most outstanding devices in diagnostics applications for screening, drug delivery, and disease identification. DEP generates a non-uniform electric field (EF) that can be applied to any biological [31] and non-biological [32], charged, or neutral particles. Particle polarization occurs as a result of charge (negative and positive) migration towards the opposite directions. The electric dipole of the particle is defined by the separation of the opposing charges [33]. This technique was first introduced by Pohl in 1951 [34]. AC field using inhomogeneous electromagnetic fields can create a trap of cells. DEP-based microfluidic has been explored to address various needs in biological, medical, and chemical areas. This technique is currently limited to the 1–10 nm accuracy [35], which is sufficient to manipulate and detect micron and nanometer-sized cells.

Cells have complex structures and consist of a cell wall, cell membrane, and nucleus with various proteins, lipid molecules, etc., making their dielectric characteristics and surface charges (i.e., electrical phenotype) of one kind unique from other types. Using the cells’ distinct differences and employing DEP electrokinetic technique, cells can be characterized, manipulated, and separated. Based on the Clausius-Mossotti (CM) function differing, the desired cells move to high EF intensity regions using pDEP and undesired cells to areas of low-intensity EFs by nDEP. This method exploits the intrinsic dielectric properties (relative permittivity and electrical conductivity) of the cell and its surrounding medium [31,32,33]. Therefore, it can be selectively used for the characterization and separation of cells. The first use of DEP for the separation of live and dead yeast cells with the aim of microbe determination was reported in 1966 [36]. In this method, they employed a rudimentary system, including a chamber with two electrodes. Later on, with microelectronics development, DEP’s application was advanced to nanometric particles such as viruses.

Interdigitated electrodes (IDEs) were the initial development in DEP microelectrodes’ miniaturization process after developing the DEP concept with wire electrodes [37]. IDE-based microfluidic devices are the most convenient DEP devices and usually consist of a microfluidic chamber or channel and IDEs patterned on the bottom surface of the channel [38]. The design of this microelectrode type is based on the two parallel adjacent bar microelectrodes with two poles. These electrodes might have equal or unequal finger width and spacing [38]. The finger width and spacing size can be in the nano to the micro-meter range [39]. The IDE-based DEP devices can be an open chamber or flow-through for cell suspension introduction or injection [40,41]. Due to the fabrication ease of the IDEs with many existing references, they are widely used in conjugation with DEP investigations. IDEs have been used to capture bacteria [42], blood erythrocytes [28], DNA [43], polystyrene beads [28,44] and capture and release ribosomal RNA (rRNA) [45]. Moreover, it was utilized to separate live and dead yeast cells [46] and Listeria innocua cells [47] and micro/nano-particles [43,48], human colon cancer cells from RBCs [49], characterize and separate C. muris, G. lambia, and C. parvum [50], and pattern colloid particles [51]. With technology development, IDEs have also been integrated with other on-chip components for different biological applications over the years. Suehiro et al. [52] developed a DEP impedance measurement (DEPIM) technique for detecting biological cells and bacteria, utilizing positive DEP force to immobilized biological particles in suspension onto an IDE array. Furthermore, IDEs are also integrated with other on-chip components for various applications. Gadish et al. [44] combined IDEs with a chaotic mixer to develop a micro-concentrator to measure the concentration of beads, B. subtilis, and spores. In other work, Vykoukal et al. [53] introduced a DEP field-flow fractionation (DEP-FFF) micro-separator with flex IDEs to enrich stem cells from enzyme-digested adipose tissue. Shim et al. [54,55] used the same DEP-FFF method for the isolation of circulating tumor cells (CTCs) from the blood.

Over recent decades, many studies have been conducted on the characterization and manipulation of the biological species on a monolithic device. Performing many functions on a single chip with a minimum amount of reagents and high efficiency increases the throughput, improves sensitivity, accuracy, and decreases operational complexity and cost. Moreover, using a single microfluidic chip allows high operation frequencies, increasing sensitivity and enabling easy mass production. Thus far, various chip materials were used for the fabrication of such a DEP microfluidic chip. Table 1 describes different DEP-on-a-chip approaches that have been tried for the fabrication of microfluidic-based LoC to enable label-free, fast, high-precision manipulation techniques and setups. Most of these systems have been integrated on various rigid and robust platforms such as silicon, glass, polymer (e.g., elastomer and PDMS), and PCB board [56,57,58,59]. For example, PDMS is one of the most common materials used for chip and microfluidic channel fabrication because it is transparent and enables optical observations. However, cost, scalability, and even ecological conditions have changed the manufacturing technologies towards disposable devices. With this background, CMOS-based LoC has been repeatedly reported as one of the most reliable platforms, capable of merging microfluidic with microelectronics, sensors, actuators, and filters as well as micro-electrochemical and microstructure systems into a monolithic device [60,61]. Compared to other DEP-on-a-chip devices, the combination of CMOS technology with DEP has been demonstrated as one of the most prominent devices in diagnostics applications for screening, drug delivery, and disease identification due to their low power consumption, scalability to larger systems like PoC, and high noise immunity [30].

Previously within our group, Guha et al. [62,63,64,65] has extensively worked on developing single CMOS LoC platforms for biological cell sensing and detection using different techniques. In all of these works, this sensor’s sensing principle is based on the relative permittivity change of the material-under-test (MUT). Variation in the biological cell resulted in the sensor’s fringing field capacity change, which caused capacitance variations to be detected by an associated silicon high-frequency read-out circuit. A non-uniform electric field between the adjacent fingers is generated by applying an electric potential to the electrodes. This method is used to detect the dielectric permittivity of the MUT. In other work [63], he proposed integrating the silicon microfluidic channel with a CMOS sensor circuit for cytometric applications to detect the cell concentrations using dielectric spectroscopy. In this method, the IDE arrays were placed on top of the microfluidic channel. As a result of IDE excitation, the fringing electric fields penetrate the fluid flowing through the channel. Other work [62] presented a CMOS-based high-frequency sensor with the capability of distinguishing the blood sample with fat and calcium from the normal blood sample. The sensor is placed at the top and inside the catheter wall and exposed to the blood.

Furthermore, he developed a self-calibrating highly sensitive dynamic IDE sensor in a BiCMOS-based PDMS microfluidic platform that can be applied for particle counting and single-particle sensing in a fluidic system [65]. The steady flow of the particles suspended in the fluid results in capacitive pulses from the sensor embedded in the oscillator. Eventually, these pulses translate to frequency modulation using an integrated phase-locked loop demodulator. They also investigated the relative viscosity variation in an aqueous solution [64] using a radio frequency (RF) CMOS chemo-bio sensor. The fringing field between the adjacent fingers is utilized to detect the dielectric permittivity of the MUT.

Several DEP integrated CMOS platforms have been developed for bio-particle characterization and manipulation (cell trapping, sorting, separation, differentiation, purification, etc.) [60,61,66,67,68,69]. However, they use relatively large-scale systems with polymeric microfluidic channels that are not compatible with the standard CMOS process flows. Silicon is a reliable replacement for polymer microfluidics in terms of high integration robustness with CMOS electronics and high precision channel alignments [70]. The bulky polymer-based LoC setups limit the device performance by introducing parasites to the system. Moreover, PDMS microfluidics is more convenient for laboratory applications than for industrial ones [71].

Here we described a CMOS-based microfluidic device that employs DEP via IDEs embedded in a silicon microfluidic channel for characterization and separation of live and dead yeast cells. This IDE platform can be integrated with circuits and microfluidic channel by the CMOS process line of IHP, for simultaneous immobilization, sensing, and detection of biological and non-biological particles. This device’s ability to be integrated with circuitry and performing many processes using a single platform for sample preparation, characterization, detection, separation, and analysis, as well as portability, makes it distinct from other setups. This study’s primary focus is to create a platform for the characterization and manipulation of cells as the preliminary step for detection and separation applications. Sensing circuitry and read-out of the cells are in the scope of this paper.

This device was successfully used to immobilize yeast cells as a means for detection application [72]. In this study, we demonstrated the applicability of the same device for frequency-dependent DEP characterization of live and dead yeast cells and for cell separation from a cell mixture. DEP responses of live and dead yeast cells were first characterized with respect to the AC field frequency and medium conductivity effects. DEP’s different polarities (pDEP and nDEP) were used to selectively separate live or dead yeast cells from a mixture of live and dead cells. Current results showed that the cell separation action was achievable using the same IDEs used for cell immobilization without any additional or specialized electrode structure. This achievement ensures maintaining the simplicity of the active devices and, as a result, the simplicity of the microfluidic chip structure. Moreover, the opportunity to employ various laboratory steps using a single device like characterization, detection, and separation makes the device analysis very prominent.

## 2. Materials and Methods

### 2.1. Microfluidic Device Design and DEP Operation

**Figure 1:**
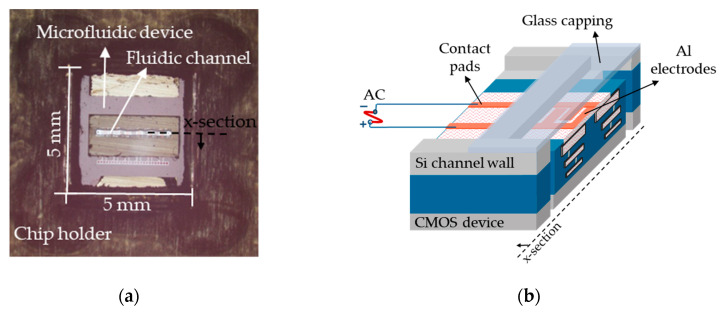
(**a**) Microfluidic device with isolated fluidic and electrical interfaces; (**b**) Schematic cross-sectional view of the device with one IDE structure.

The DEP microfluidic device used in this work was introduced in our recent article [72] for cell immobilization. To analyze biological suspensions, a CMOS device was combined with a microfluidic channel on a single chip. This device is composed of a CMOS integrated microfluidic channel with embedded Al-based IDEs at the bottom of the silicon channel. For simultaneous electrical measurement and optical observations, the microfluidic channel was closed with a glass layer. The fabrication was performed based on the standard 250 nm high-performance SiGe BiCMOS technology of IHP. Figure 1a shows the microfluidic device, which consists of one channel with 2.75 mm length, 0.33 mm width, and 0.075 mm depth with two ports for the inlet and outlet. There are six multi-fingered IDEs with various geometrical dimensions, spaced 250 µm apart, fabricated on the topmost metal level of the back-end-of-line stack of the CMOS process. Figure 1b presents the schematic cross-sectional view of the microfluidic device. The fabrication details and procedures have been previously explained in [72,73]. The microfluidic channel delivers the liquid to the electrodes, and the IDEs model the DEP structures. In this work, for separation experiments, we focused on the results achieved from one of the best operational IDEs [72,74]. The finger width and gap of this IDE are 45 µm and 5 µm, respectively.

**Figure 2:**
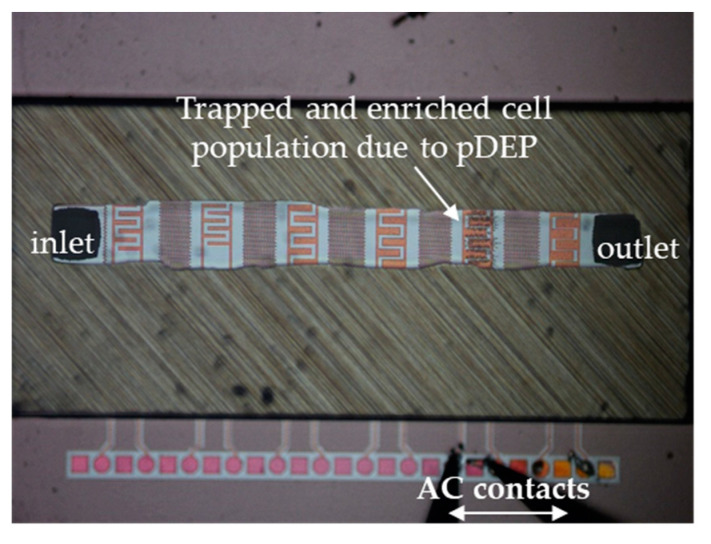
Microfluidic device in operation: cell entrapment at the IDEs after imposing AC and generating DEP.

For cell characterization, the microfluidic channel was first filled with either live or dead cell suspensions, and then for the separation experiments media was replaced by live and dead cell mixture suspension. The liquid suspension was generated in the microfluidic channel through the inlet with 1 µm s^−1^ flow rate, where the IDEs are present to induce the DEP effect by imposing the AC signal (Figure 2).

Cells experienced positive DEP (pDEP) attracted toward the IDEs and trapped there. Meanwhile, in separation operations, cells that undergo negative DEP (nDEP) did not overcome the hydrodynamic drag force to be immobilized at the IDEs and washed away from the channel in the stream of fluid flow. This, resulted in the enrichment of one cell kind from the other one.

### 2.2. Sample Preparation

Cells of Saccharomyces cerevisiae RXII, Commercial fresh baker’s yeast, were used in this study. The samples were suspended in four various medium solutions with different conductivities for DEP experiments. These medium were such as DI-water (DIW), Tap water, Potassium chloride (KCL) solution (Supelco, Merck KGaA, Darmstadt, Germany), Phosphate-buffered saline (PBS) solution (Sigma-Aldrich, Merck KGaA, Darmstadt, Germany), and diluted PBS (D-PBS) with DIW in a volume ratio of 1:25. These suspending mediums were used to compare the DEP behavior of the cells. The choice of media was made so that conductivities of different orders of magnitude ranging from 10^−4^ to 1 were investigated. Once solutions were prepared with desired dilutions, their conductivities were measured at room temperature using a conductivity meter (Accumet AET30 Conductivity Tester, Thermo Fisher Scientific, Inc., Waltham, MA, USA). The yeast cells were considered to be robust enough to survive any osmotic stress that can be caused by the non-physiological media used. To prepare stock yeast suspensions, 30 mg of baker’s yeast (fresh form) was diluted in 20 mL of medium solutions. The samples were equally transferred into 2mL micro centrifuge tubes. To achieve dead cells, a yeast sample tube was heated at 100 °C for 30 min in a dry bath block (AccuBlock Mini-Compact Dry Bath, Labnet International, Inc., Edison, NJ, USA).

For characterization, ten suspensions were prepared and applied separately. To this end, live and dead cells were individually suspended in 0.0002 S/m DIW, 0.08 S/m Tap water, 0.2 S/m KCL 20 mM, 1.39 S/m PBS 0.1 M and 0.1566 S/m D-PBS 0.1 M solutions. The PH values of the DI-water, tap-water, KCL, PBS, and D-PBS were measured 7, 7.04, 7.05, 7.8, and 7.1, respectively, using PH meter (Checker Plus, Hanna Instruments Deutschland GmbH, Germany). For separation, live and dead cell suspensions were mixed in a 1:1 ratio and stained with methylene blue (MB) dye (Sigma-Aldrich, Merck KGaA, Darmstadt, Germany). Due to the high cell concentration of cell mixture suspension, the prepared stock solutions were further diluted with their respective medium solutions in the volume ratio of 1:10 for separation experiments, cell density, and the viability test measurements. The 4 mL of 1:10 cell mixtures were stained with 1 mL MB for 20 min.

The induced DEP force by the IDE electrodes was generated by AC voltage (V) supplied by a signal generator (Agilent-33220A, Agilent Technologies/Keysight Technologies, Santa Clara, CA, USA). Cell tracing and motions were monitored and recorded by a CCD camera (Nikon-DS-Fi2, Nikon GmbH, Tokyo, Japan) mounted on an optical microscope (Nikon Eclipse-LV100ND, Nikon GmbH, Tokyo, Japan). Cell suspension solutions were pumped through the channel using a syringe pump (NEMESYS, CETONI GmbH, Korbußen, Germany). A hemocytometer (BLAUBRAND^®^ Neubauer improved, BRAND GMBH + CO KG, Germany) was used to estimate the cell viability and density by counting the cells per volume concentration unit (cells/mL). The viability of the cell mixture was determined using a counting chamber (hemocytometer) through optical microscope observation. The heat-killed cells were stained and showed a blue-color cytoplasm, and, conversely, live cells were not stained. Therefore, the blue spheres represent dead cells, while the white ones represent live cells. Counting the ratio of the viable cells to the total number of the cells gives an estimation about the viability of the sample before and after the measurement.

The cellular density of the stock solutions were estimated to be in the average range of $1.45\times 10^{7}$ cells mL^−1^. The cellular density of the mixtures was estimated to be in the average range of $1.3\times 10^{5}$ cells mL^−1^. The conductivity value of the live and dead yeast suspension samples is shown in Table 2.

## 3. Theoretical Background

### 3.1. Dielectrophoresis Theory

Dielectrophoresis (DEP) is the motion of a polarizable cell in a dielectric medium subjected to a non-uniform AC electric field [1]. The magnitude of the induced dipole depends on the polarizability of the cell and the surrounding media. The DEP force on a spherical cell of radius *r* is defined as:

(1) $$F_{DEP}\left(t\right)=2\pi \varepsilon_{m}r^{3}\;Re\left[f_{CM}\right]\;\nabla |E_{rms}{|}^{2},$$

here $\varepsilon_{m}$ represents the relative permittivity of the suspending media, $Re\left[f_{CM}\right]$ is the real part of the Clausius-Mossotti factor ($f_{CM}$), and $\nabla |E_{rms}{|}^{2}$ is the root-mean-square of electric field strength and is related to the voltage V. The $f_{CM}$ is a complex number:

(2) $$f_{CM}=\frac{\varepsilon_{c}^{*}-\varepsilon_{m}^{*}}{\varepsilon_{c}^{*}+2\varepsilon_{m}^{*}},$$

(3) $$\varepsilon^{*}=\varepsilon -i\frac{\sigma }{\omega },$$

where $\varepsilon_{m}$ represents the relative permittivity of the suspending media, $\varepsilon_{m}^{*}$ is the complex permittivity of the fluid and, $\varepsilon_{c}^{*}$ is the complex permittivity of the cell. Complex permittivity is the function of the conductivity (*σ*) and angular frequency (*ω*) of the electric field. The electric field relationship illustrates the local electric field E, which is linked to the potential field V:

(4) E=−∇V

The intensity of E at a point is the gradient of potential V at that point after sign change, in the x-direction.

As can be seen from (1), DEP force is strongly dependent on the cells’ size, electrical and dielectric properties, their surrounding media ($f_{CM}$), voltage, frequency, and electric field vectors. When $\sigma_{p}<\sigma_{m}$ and $\varepsilon_{p}$ > $\varepsilon_{m}$, $f_{CM}$ is negative at lower frequencies and positive at higher frequencies, and when $\sigma_{p}$ > $\sigma_{m}$ and $\varepsilon_{p}<\varepsilon_{m}$, $f_{CM}$ becomes positive at lower frequencies and negative at higher frequencies. Therefore, the positive and negative values of $f_{CM}$ results in either positive DEP (pDEP) or negative DEP (nDEP), respectively. When $Re[f_{CM}]>0$, cells attract toward the high electric field intensity regions. In contrast, when $Re[f_{CM}]<0$, cells repel from these regions. The transition point from positive to negative or vice versa is known as crossover frequency (fc) or zero force-frequency [75]. This is a specific point at which the real part of the cell’s effective polarizabilities and its suspending media are equal (i.e., $Re[f_{CM}]=0$), thus making the DEP force zero. Creation of the electric field gradient in each of the induvial cells as a result of their electric dipole can cause the attraction of the cells to their neighboring cells. Therefore, the polarized cells creates pearl-chains that are formed along the electric field lines [76]. Based on the selective DEP forces, desired cells can be trapped and isolated from a cell mixture for purifying processes [25]. Furthermore, dead cells can be removed from live cells [23].

Applying AC to these electrodes, a non-uniform EF is created. This EF is the strongest at the finger edges and the weakest in the adjacent finger gaps (i.e., electrode intervals) and the electrode finger’s center. The field’s magnitude is decreased with the height from the electrode surface, and thus DEP force is significantly reduced in a vertical direction. Therefore, the DEP force is inversely proportional to the distance from the IDEs generating EF [29]. Depending on the relative permittivity of the cells and medium flown over IDEs as well as amplitude and angular frequency of the AC, cells can experience a translational DEP force in two opposite directions. Positive DEP force moves the cells toward the strongest EF locations (i.e., finger edges), and as a result, cells can be trapped there. Negative DEP repels the cells from the IDEs and moves them towards the lowest EF regions.

### 3.2. Numerical Determinations of Clausius-Mossotti Factor

**Figure 3:**
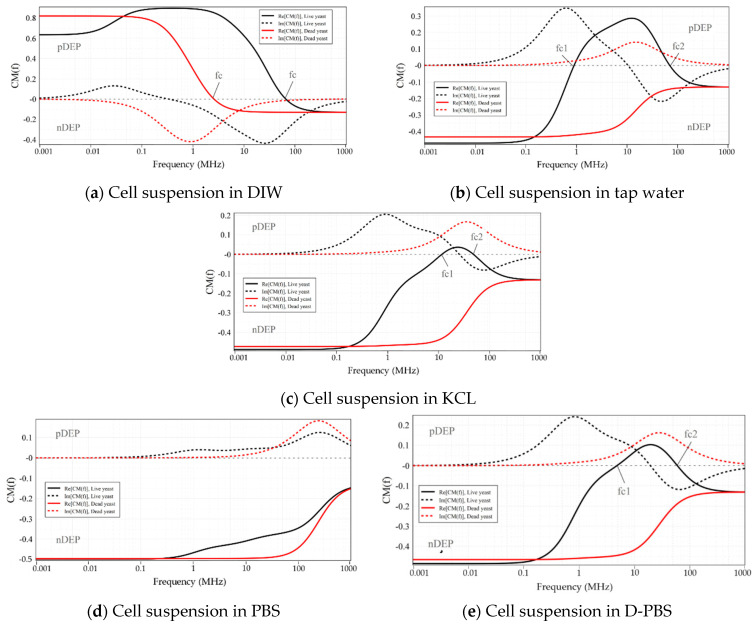
The Clausius-Mossotti factor as a function of frequency for live and dead yeast cells suspended in: (**a**) DIW; (**b**) Tap-water; (**c**) KCl; (**d**) PBS; (**e**) D-PBS. Electrical and geometrical properties of the live and dead yeasts were taken from [79]. Solid lines and dotted lines represent the real and imaginary parts of the Clausius-Mossotti factor, respectively.

Figure 3 illustrates the CM factor’s ovulation with the frequency of the applied electric field for live and dead yeasts suspended in DIW (0.0002 S/m), Tap-water (0.08 S/m), KCL solution (0.2 S/m), PBS (1.39 S/m), and D-PBS (0.1566 S/m). The numerical determinations were calculated using myDEP software [77], based on the two-shell model [78], where cells are assumed to possess two concentric layers of various electric and dielectric properties.

Based on these calculations, as shown in Figure 3a, the CM factor’s real part of live cells suspended in DIW was bounded between 0.9 and −0.13. For dead cells, this value was specified between 0.82 and −0.13. For cell suspension in DIW fc was predicted at around 65.4 MHz and 2.5 MHz for live and dead yeast, respectively. At frequencies lower than these crossover frequencies, the DEP force was positive on both live and dead cells. For live-cell suspension in tap-water (Figure 3b) CM factor was bounded between 0.28 and −0.47. In contrast, for dead-cell suspension $f_{CM}$ was bounded entirely in the negative region. For the KCL suspending media (Figure 3c), with a dilution of 20 mM, the same trend as tap-water can be seen for the live and dead cells. For both live and dead cells suspended in tap-water and KCL, the maximum value of the CM factor for nDEP was around −0.49, whereas that for pDEP was around 0.28 and 0.03, respectively. This indicates that forces generated by pDEP are weaker than nDEP forces. For KCL compared with the tap-water, not only the maximum value of CM factor was less, but also the pDEP spectrum was limited to smaller frequency ranges. Figure 3d, shows the CM factor for the highest conductance suspending media (PBS, 0.1M), where the real part for both live and dead cells was under nDEP for all frequencies. However, for diluted PBS live-cell suspension, a pDEP spectrum is expected over a more comprehensive frequency range than KCL (Figure 3e). For this cell suspension the real part of $f_{CM}$ was between 0.1 and 0.48.

## 4. Results and Discussion

**Figure 4:**
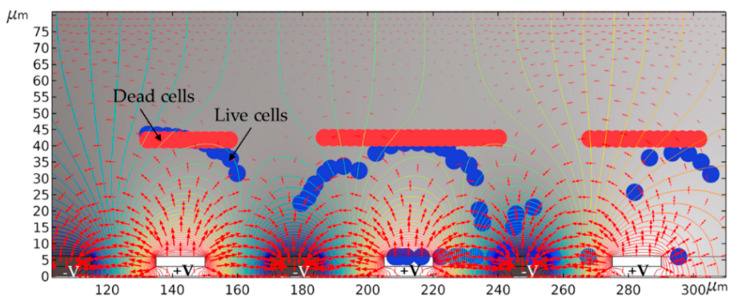
FEM simulation results for cell separation. DEP generated by IDEs, shown in black and white segments (marked by −V and +V, respectively). The line contour illustrates the electric potential applied to the IDEs, and red arrows represent the electric field distribution.

The DEP separation of cell mixtures using the same operating conditions and experimental configurations was simulated using COMSOL Multiphysics^®^. For these simulations, we used the same model described in our previous publication [73]. The related parameters and boundary conditions are explained in detail in [72,73]. Figure 4 illustrates the DEP isolation of live cells from dead cells suspended in KCL at 20Vpp, 6 MHz, and 1 µm s^−1^.

As it was expected from the calculations, live cells experienced pDEP and attracted by the higher electric field intensity regions, and finally got trapped at the IDEs. Meanwhile, dead cells were not influenced by pDEP and moved towards the lower electric field intensity regions, which eventually led to their elution from the channel and separation from the live cells.

### 4.1. Characterization of Live and Dead Yeast Cells

**Figure 5:**
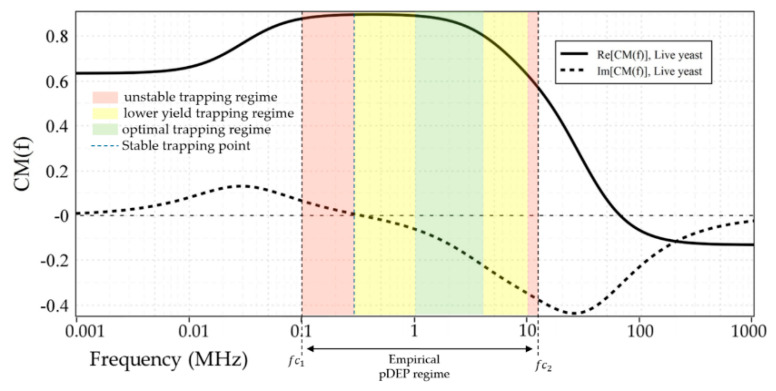
Empirically proven pDEP regime and crossover frequencies (*fc*) vs. numerical predictions for live yeasts suspended in DIW. Colored frequency bands represent trapping behavior based on microscopical observations.

The frequency-dependent DEP behavior of the live and dead cells was first determined empirically by observing cells’ behaviors when the given frequency was altered to see whether cells move towards the IDEs or away from them. To this end, 20 Vpp was applied with ranging frequencies between 10 kHz to 20 MHz (maximum frequency allowed by the signed generator). As the numerical calculations predicted, both live and dead yeasts suspended in DIW exhibited pDEP and nDEP behaviors. Figure 5 compares the numerical predictions and the empirically proven pDEp regime. Results showed that, contrary to CM factor calculations, the pDEP spectrum for live yeasts in DIW was restricted between 100 kHz–12 MHz. The frequency range at which cells experienced enough DEP force to get trapped was between 300 kHz–10 MHz for live cells, and the experimental fc point was different from the numerical predictions.

**Figure 6:**
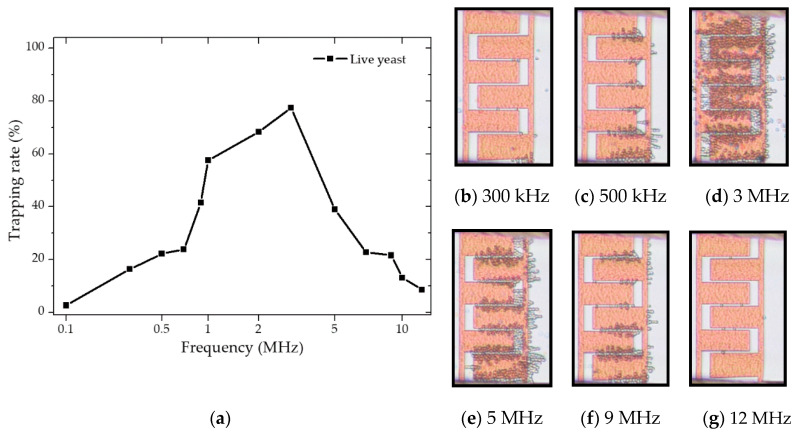
DEP characterization of live yeasts in DIW at 1 µm s^−1^ flow rate: (**a**) Trapping rate (yield) approximation by changing frequency based on covered cell area at the IDEs; (**b**–**g**) the images show the trapping at various frequencies after 1 min.

Moreover, within a specific frequency range (green region), the trapping yield was optimum. The trapping yield was lower in yellow regimes than in the green areas. In the pink regions, unstable trappings were observed. Upon applying these ranges, entrapped cells tended to desorb from the electrodes after few seconds. The blue dashed line represents the point at which trapping was getting stable, and desorption of entrapped cells reached the minimum. These variations were observed by the microscopical magnification. Several images were taken at various steps to analyze and estimate the trapping yield for given frequencies. The images were then analyzed using ImageJ software. Each picture had a specific cell coverage on the entire IDE area. The trapping percentage was estimated by calculating the area covered by cells to the total area, as demonstrated in Figure 6a. These measurements were done at the frequency range of 10 kHz–20 MHz. For these live cells, the optimum frequency range was in the field of 1–3 MHz. Figure 6b–g describes the trapping behavior upon changing frequency. For frequencies below 300 kHz and beyond 10 MHz (within the mentioned frequency band), the DEP force could only impact the cell trajectory and not trapping.

It was observed that the velocity reduction of cells reaching the IDEs due to partial absorption toward the IDE region was followed by spontaneous desorption. This could be related to an insufficient amount of pDEP force to trap the cells stably at the IDEs. Upon changing frequency and applying frequencies between 300 kHz–10 MHz, cell trapping got stable, and optimal trapping was achieved between 1 to 3 MHz.

**Figure 7:**
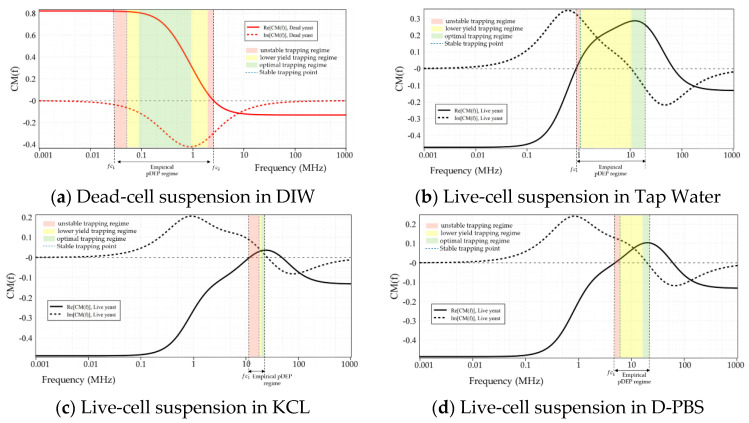
Empirically proven pDEP regime and crossover frequencies (*fc*) vs. numerical predictions for: (**a**) dead yeast suspension in DIW and live cell suspensions in; (**b**) Tap water; (**c**) KCL; (**d**) D-PBS. Colored frequency bands represent empirically proven trapping behavior based on optical observations.

DEP characterization measurements on dead yeast suspensions showed that the positive scope for dead yeasts in DIW media was between 20 kHz–2.3 MHz. The frequency at which the fc occurred (2.3 MHz) was in good agreement with the numerical simulation results. However, the frequency range at which cells experienced enough DEP force to get trapped was between 70 kHz–2.3 MHz. As shown in Figure 7a, the empirically proven pDEP regime of dead cells occurred at lower frequencies and over a narrower frequency band than live cells. Further observations indicated that the live cells suspended in tap-water and KCL experienced both negative and positive DEP.

As expected from simulations, live yeast cells in PBS solution experienced nDEP, whereas, in diluted PBS, both negative and positive forces acted on the live cells. Dead cell dilutions in tap-water, KCL, PBS, and D-PBS solutions were solely under nDEP over the entire frequency range. Due to our signal generator’s limitation, which was restricted to 20 MHz, investigation of the complete range of pDEP was not possible for all of the live cell suspensions. However, for all the cases, fc1 matched closely with the numerical analysis. DEP force-frequency profile indicated that, keeping all the other parameters constant, changing frequency influences the cell trajectory and gives rise to DEP force in two directions (positive and negative) and impacts the trapping rate (yield) at the IDEs. The trapping trend for all the cases was almost similar. They exhibited a gradual increase in trapping rate by the transition of the real part from negative to positive, and the trapping rate gained peak by increasing frequency. It was seen that the smaller the cell size is, the lower frequency ranges are required for trapping. At lower frequencies, larger electric field gradients were necessary for effective DEP trapping and manipulation. Thus, trapping efficiency can be tuned with increasing electric potential.

**Figure 8:**
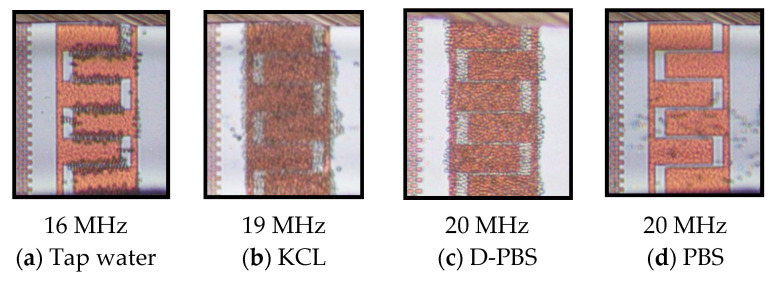
DEP behavior of live yeasts in different medium solutions at 20 Vpp and 1 µm s^−1^ flow rate: (**a**–**c**) pDEP, which leads to cell trapping and; (**d**) nDEP and no cell trapping.

Figure 8a–c demonstrates the trajectory of live cells suspended in various solutions (tap water, KCL, PBS, and D-PBS), leading to cell entrapment at the IDEs except for cell suspension in PBS (Figure 8d) that no trapping occurred due to nDEP and cell continued moving in the stream of fluid flow.

Comparing all the results, it can be concluded that as the medium conductivity increases, the pDEP spectrum gets smaller, and trapping occurs at higher frequencies. The trapping trend for all the cases was almost similar. The trapping yield gradually increased by the transition of the real part from the negative (dominant conductivity region) to positive (where permittivity starts to dominate) and gained peak with increasing frequency. Moreover, the trapping yield is maximized when $Re\left[f_{CM}\right]$ achieves its peak values. Table 3 summarizes the DEP characterization results of viable and dead cells diluted in various medium solutions.

### 4.2. Cell Separation from a Cell Mixture

**Figure 9:**
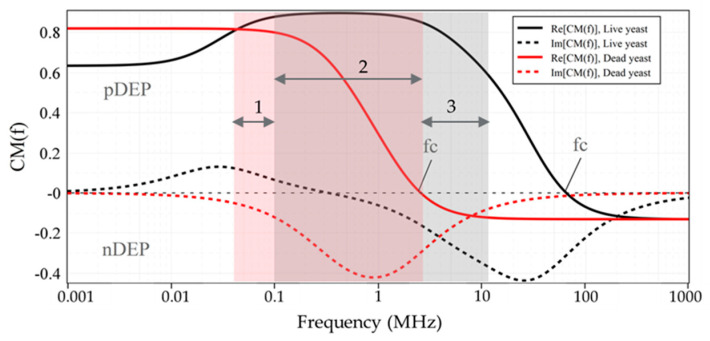
Experimentally measured (pink and gray zones) and numerically predicted (lines) affective pDEP for live and dead cells in 0.0002 S/m DIW at different AC field frequencies at the microfluidic channel using pDEP.

Based on numerical predictions and characterization measurements, it was evaluated that each of the live and dead cells has distinct DEP responses with respect to their surrounding media. Considering these particular behaviors, we used the same microfluidic device to separate and isolate a cell type from a cell mixture. Figure 9 combines the experimentally measured effective pDEP ranges for both live (highlighted gray zone) and dead (highlighted pink zone) cells in DIW with the numerical predictions into one plot. Such a graph can be used as a phase diagram for separation regimes of the cell mixture at the microfluidic channel. The labeled 1–3 regions specify the effective frequency ranges for continuous separation of either live or dead cell types.

Upon applying any frequencies from zone 1, only the dead cell can get trapped and isolated from the live cells. In contrast, zone 3 was the region where only live cells can be trapped and isolated from the dead cells, while dead yeasts can flow through the channel and be separated from live ones. Upon switching to any frequencies from zone 2, i.e., the highlighted overlapped region, both cell types can get trapped. Therefore, to selectively trap live or dead cells from the mixture, frequencies less than 100 kHz (in zone 1) and more than 2.3 MHz (in zone 3), respectively, were the most effective conditions for differential separation. The cells leaving the microfluidic channel can be collected at the outlet and used for further investigations and analysis, such as viability test.

**Figure 10:**
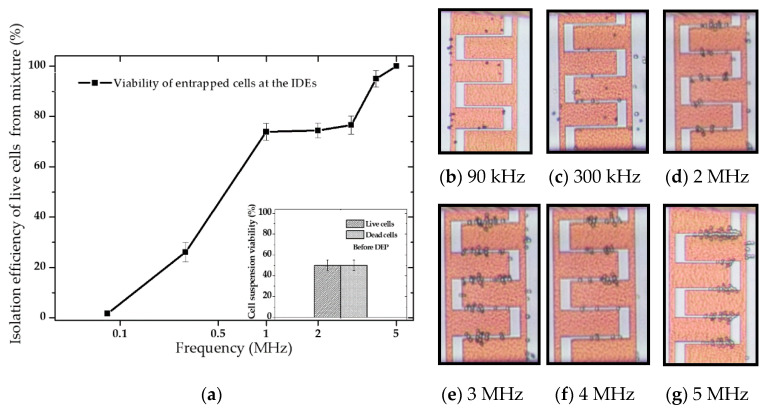
Experimentally recorded results on selectively trapping and separation of live cells from a live and dead yeast mixture (0.1282 S/m) suspended in DIW at a constant voltage of 20 Vpp: (**a**) Isolation efficiency of live cells from a mixture using IDEs; (**b**–**e**) Micrograph examples where the percentage of isolated live cells increase by increasing frequency (**b**) 0%; (**c**) 22.22%; (**d**) 77.35%; (**e**) 80.19%; (**f**) 91.66%; and (**g**) 100%. White cells are Live, and blue-dyed cells are dead cells. The live and dead cells mixture was 1:1 in the suspension prior to separation.

The isolation efficacy of live cells from a cell mixture was determined during the DEP process for frequencies within zone 1–3, as represented in Figure 10a. This was performed by instantly calculating the viability percentage of entrapped and isolated cells at the IDEs via live and dead cell counting. Viability is the ratio of the number of live (white) cells to the total number of entrapped live and dead (blue-dyed) cells. By switching frequency, the targeted cells were isolated at the IDEs from the cell mixture introduced into the microfluidic channel using the 1 µm.s^−1^ flow rate at 20 Vpp. Upon applying frequencies from zone 1, only dead cells could be isolated (Figure 10b). By increasing to frequencies from zone 2, both live and dead cells were trapping, Figure 10c–f. However, the yield of the entrapped live cell population was increasing steadily compared to dead cells, and the isolation efficiency of the live cell population reached 100% at around 5 MHz (Figure 10g). The shift of the higher crossover frequency of dead cells (from 2.3 MHz to about 4.5 MHz) can be attributed to the medium solution’s conductivity elevation (from 2.23 × 10^−3^ S/m to 0.128 S/m) due to staining. Applied frequencies below 100 kHz (within zone 1) diminished the isolation efficiency of live cells drastically to 0%, where the isolation efficiency of dead cells reached 100%.

**Figure 11:**
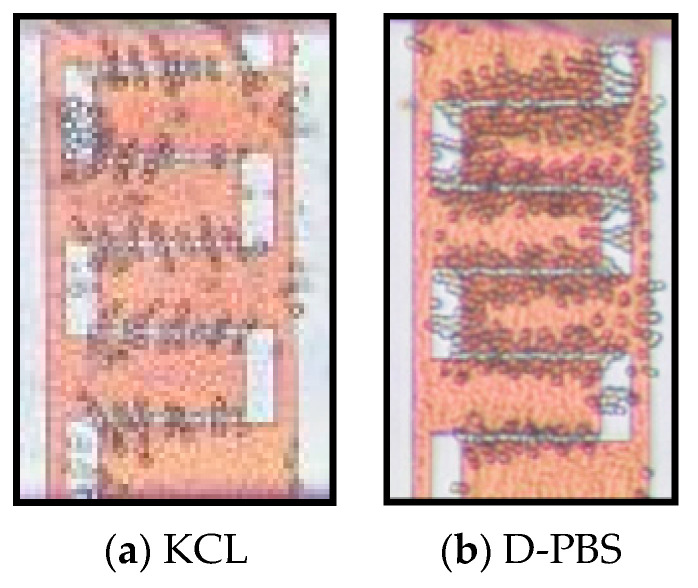
Differential separation of the live yeasts from live and dead (1:1) cell mixtures by DEP at 20 V_pp_, 20 MHz while dead cells were removed by nDEP: (**a**) cell mixture in KCL; (**b**) cell mixture in D-PBS.

Figure 11 demonstrates the differential separation of live cells from live and dead mixtures in KCL and in D-PBS with almost 100% isolation efficiency at 20 MHz.

**Figure 12:**
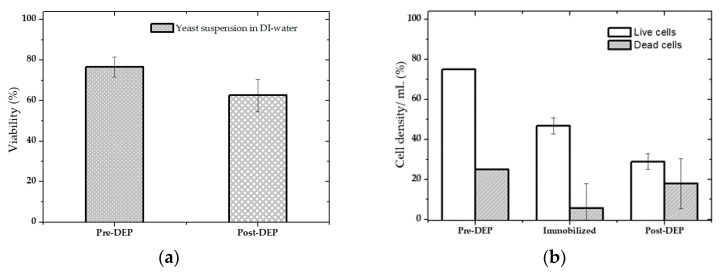
Separation estimation of live/dead yeasts in a mixture. The live and dead cells mixture was 3:4 in the suspension before separation: (**a**) Viability estimation before (Pre-DEP) and after measurement (Post-DEP); (**b**) Percentage of trapped and released live and dead cell densities after DEP. The separation condition was: 20 Vpp, 1MHz, and 1 µm·s^−1^ flow rate.

As demonstrated in Figure 12a, analysis before and after DEP showed that the cell solution’s viability dropped by 14% after DEP. This viability test approximation showed that at 1 MHz (which was in the overlapped region—zone 2), live yeasts experienced the pDEP force more than the dead yeasts. As a result, more live cells were trapped and isolated from the mixture (see Figure 12b).

It was estimated that upon separation, more than 59% of the live cells and 37% of the dead cells remained in the pDEP at the IDEs when almost 63% of the dead yeasts and less than 41% of live ones were removed from the channel. This is because dead cell response to pDEP is weaker due to the lack of permittivity and conductivity difference with its surrounding buffer. Furthermore, at 1 MHz, the DEP force is closer to fc and weaker than lower frequencies in its effective pDEP region. As a result, the release of dead cells from the channel is 20% more than live ones.

### 4.3. Isolation Efficiency Assay

**Figure 13:**
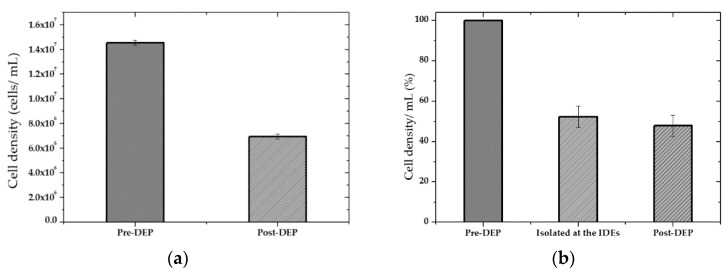
Cell density assay before and after DEP. The cells were immobilized using pDEP at 1 MHz, 20 V_pp_: (**a**) Approximate cell density before (Pre-DEP) and after measurement (Post-DEP); (**b**) Percentage of trapped and released cell density after DEP.

For yeast suspension with high cell density in DIW, the isolated cells’ efficacy at the IDEs was approximately estimated at 20 Vpp, 1 MHz, via counting of the cells before DEP and after DEP when the AC was still on. 1 mL cell suspension was introduced in the microfluidic channel using a flow rate of 1 μm·s^−1^. The remaining cells that were not trapped at the IDEs and released from the channel were collected in the tubes at the outlet for further analysis (counting and viability test). Figure 13a shows the cell density assay before and after DEP. Using these approximations, the percentage of the entrapped cells was calculated by deducting the number of the collected cells at the outlets (Post-DEP) from the total number of the cells (Pre-DEP), Figure 13b. 

Analyses estimations, for high cell density sample, showed that upon DEP, almost 53% of the cells were trapped at the electrodes. In contrast, around 47% did not overcome drag force and were washed and released from the channel since they were either affected very slightly by pDEP force or did not experience at all. This can be related to the height of the microfluidic channel compared to the cell size and the effective distance of the electric field gradient over the electrodes or the orientation of IDEs in the microfluidic channel. The electric field’s magnitude over the IDEs decays with the distance over the IDEs towards the top of the microfluidic channel. Therefore, cells flowing from significantly above the effective electric field region cannot get affected by the electric field gradient because they do not encounter this field and are deflected by any of the DEP forces and only kept flowing in the solution stream eluted from the microfluidic channel. The DEP force decreases quickly as the distance from the planar electrodes increases. Thus, this can likely be improved upon reducing the channel height (75 µm) by at least half of its current size. Moreover, as cell loading continued during the DEP process, the IDEs started to saturate with entrapped cells, and pearl chains structure were formed. This causes a decrease in the trapping yield because the pDEP areas became unavailable to new cells. On the other hand, oversaturation occurred, which led to a significant number of cells desorption with the time passage. However, letting the cells be settled for a more extended period when they reached the channel or adjusting the sample’s cell density to lower orders of magnitude (<$10^{6}$) improved the trapping efficiency (>99%).

Experimental results proved that our microfluidic device has a very fast response, and the trapping starts in a short time after applying AC potential. At optimum AC ranges, trapping occurs spontaneously, in less than 1 s, as soon as the AC was used. Depending on other applied AC ranges, this time increased to 5 s after running the fluid through the microfluidic channel. Cutting the AC, desorption arose very fast.

## 5. Conclusions

A CMOS integrated silicon-based microfluidic device with embedded arrays of IDE was presented for characterization and separation of live and dead cells and selective separation of one type from the other type in a mixture. This microfluidic device has been used to characterize the DEP responses of these cells in terms of positive and negative DEP and crossover frequency. Various cell suspensions were used during the measurements, and the results were compared with the numerical simulations. Moreover, the selective separation of live yeast cells from dead yeast cells was demonstrated using this device. The differential separation was found to work sufficiently well to be suited for implementing the final design. This device is suitable for the analysis of cells suspended in media with conductivity not more than 10^−1^ S/m order of magnitude. Results showed that as the medium conductivity increases, the pDEP spectrum gets smaller, and trapping occurs at higher frequencies.

Moreover, it was observed that by increasing conductivity, the crossover frequency (transition point of real part from negative to positive) was increased. For highly conductive media (such as PBS), no pDEP response was observed over the cell trajectories, and as a result, no cell entrapment was achieved. However, pDEP trapping and cell separation were tunable for such a buffer solution by changing the medium’s electrical conductivity.

## Figures and Tables

**Table 1:** Various DEP-on-a-chip platform examples for cell analysis applications.

Ref.	Purpose	Analyte	Microfluidic Material	DEP Microelectrode	DEP Parameters	Substrate
[56]	characterization, identification	Stem cells and their differentiated progeny	PDMS	IDE ^1^	25 kHz—10 MHz at 8 V	silicon wafers or glass slides
[57]	detection	Live Jurkat’s cytoplasm	PDMS	coplanar waveguide (CPW)	10 MHz at 3 V	quartz
[58]	trapping, rotating, detecting	Hela cells and polystyrene particles	PDMS	IDE ^1^	1 MHz at 76/80 Vpp	PCB
[59]	detection, separation	Micro-nano particles 500 nm–10 µm	Glass	L-shaped electrode	0–1 MHz at >−1.3 V and <1.4	glass on PCB
[60]	trapping, manipulation	yeast cells	PDMS	3D octa-pole	100 Hz—5 MHz at 5 Vpp	CMOS
[61]	characterization, discrimination	cancer stem cells	PDMS	quadrupole electrode	50–500 MHz at 2–4 Vpp	CMOS

^1^ Interdigitated electrode.

**Table 2:** Conductivity value of cell suspension samples.

Sample	DI-Water	Tap Water	KCL	PBS	D-PBS
Live yeast cells	1.18 × 10^−3^ S/m	7.82 × 10^−2^ S/m	0.262 S/m	1.430 S/m	0.201 S/m
Dead yeast cells	3.87 × 10^−3^ S/m	7.85 × 10^−2^ S/m	0.266 S/m S/m	1.435 S/m	0.206 S/m

**Table 3:** DEP characterization results for yeast cell suspensions.

MUT ^1^		pDEP Range (Numerical Prediction)		pDEP Range (Empirical)		
Cell	Media	fc1 ^2^	fc2	fc1	fc2	f_opt_^3^
Live Yeast cells	DIW	-	65.4 MHz	100 kHz	12 MHz	1–3 MHz
	Tap water	887 kHz	68.2 MHz	1 MHz	40 MHz ^4^	10–14 MHz
	KCL (20 mM)	11.6 MHz	48 MHz	11 MHz	not measured	18–20 MHz
	PBS (0.1 M)	nDEP	nDEP	nDEP	nDEP	-
	D-PBS	4.86 MHz	59.98 MHz	4 MHz	not measured	13–20 MHz
Dead Yeast cells	DIW	-	2.5 MHz	20 kHz	2.3 MHz	90–900 kHz
	Tap water	nDEP	nDEP	nDEP	nDEP	-
	KCL (20 mM)	nDEP	nDEP	nDEP	nDEP	-
	PBS (0.1 M)	nDEP	nDEP	nDEP	nDEP	-
	D-PBS	nDEP	nDEP	nDEP	nDEP	-

^1^ Material under test, ^2^ Low-and high crossover frequency points, ^3^ Optimum trapping frequency range, ^4^ Measured through separate experiments not mentioned in the text.
